# Loss of TAZ after YAP deletion severely impairs foregut development and worsens cholestatic hepatocellular injury

**DOI:** 10.1097/HC9.0000000000000220

**Published:** 2023-08-09

**Authors:** Adelya Gabdulkhakova, Yekaterina Krutsenko, Junjie Zhu, Silvia Liu, Minakshi Poddar, Sucha Singh, Xiaochao Ma, Kari Nejak-Bowen, Satdarshan P.S. Monga, Laura M. Molina

**Affiliations:** 1 Precision Digital Health, Department of Cardiology, Angiology and Pneumology, University Hospital Heidelberg, Germany; 2 Department of Pathology, Division of Experimental Pathology, University of Pittsburgh School of Medicine, Pittsburgh, Pennsylvania, USA; 3 Department of Pharmaceutical Sciences, Center for Pharmacogenetics, University of Pittsburgh School of Pharmacy, Pittsburgh, Pennsylvania, USA; 4 Pittsburgh Liver Research Center, University of Pittsburgh Medical Center, Pittsburgh, Pennsylvania, USA; 5 Department of Medicine, Division of Gastroenterology, Hepatology, and Nutrition, University of Pittsburgh School of Medicine, Pittsburgh, Pennsylvania, USA; 6 Medical Scientist Training Program, University of Pittsburgh School of Medicine, Pittsburgh, Pennsylvania, USA

## Abstract

We previously showed that loss of yes-associated protein 1 (YAP) in early liver development (YAP^KO^) leads to an Alagille syndrome-like phenotype, with failure of intrahepatic bile duct development, severe cholestasis, and chronic hepatocyte adaptations to reduce liver injury. TAZ, a paralog of YAP, was significantly upregulated in YAP^KO^ hepatocytes and interacted with TEA domain family member (TEAD) transcription factors, suggesting possible compensatory activity.

**Methods:**

We deleted both *Yap1* and *Wwtr1* (which encodes TAZ) during early liver development using the *Foxa3* promoter to drive Cre expression, similar to YAP^KO^ mice, resulting in YAP/TAZ double knockout (DKO) and YAP^KO^ with TAZ heterozygosity (YAP^KO^ TAZ^HET^). We evaluated these mice using immunohistochemistry, serum biochemistry, bile acid profiling, and RNA sequencing.

**Results:**

DKO mice were embryonic lethal, but their livers were similar to YAP^KO^, suggesting an extrahepatic cause of death. Male YAP^KO^ TAZ^HET^ mice were also embryonic lethal, with insufficient samples to determine the cause. However, YAP^KO^ TAZ^HET^ females survived and were phenotypically similar to YAP^KO^ mice, with increased bile acid hydrophilicity and similar global gene expression adaptations but worsened the hepatocellular injury. TAZ heterozygosity in YAP^KO^ impacted the expression of canonical YAP targets *Ctgf* and *Cyr61*, and we found changes in pathways regulating cell division and inflammatory signaling correlating with an increase in hepatocyte cell death, cell cycling, and macrophage recruitment.

**Conclusions:**

YAP loss (with or without TAZ loss) aborts biliary development. YAP and TAZ play a codependent critical role in foregut endoderm development outside the liver, but they are not essential for hepatocyte development. TAZ heterozygosity in YAP^KO^ livers increased cell cycling and inflammatory signaling in the setting of chronic injury, highlighting genes that are especially sensitive to TAZ regulation.

## Graphical abstract

**Figure:**
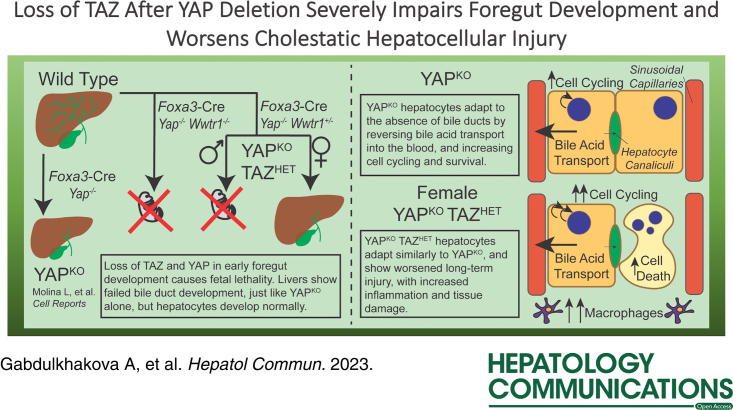

## INTRODUCTION

Yes-associated protein 1 (YAP) is a transcriptional coactivator that partners with transcriptional enhanced associate domain (TEAD) domain family member transcription factors to regulate critical functions in liver development, regeneration, and tumorigenesis.^1,2^ Previously, we interrogated the function of YAP in early liver development by studying a mouse model in which YAP was deleted from the foregut endoderm between embryonic days 8–12 using *Foxa3* promoter–driven expression of Cre-recombinase (YAP^KO^ mouse).^3^ Loss of YAP in liver progenitors resulted in the early arrest of bile duct development, causing the absence of functional bile ducts in YAP^KO^ livers. Incredibly, these mice survived and had relatively long lifespans, displaying significant adaptations in the liver to prevent bile acid toxicity and altering the balance between metabolic and regenerative pathways to promote injury repair. Bile acids were secreted into the bloodstream rather than the hepatocyte canaliculi and were more hydrophilic to reduce toxicity.^3^

**FIGURE 1:**
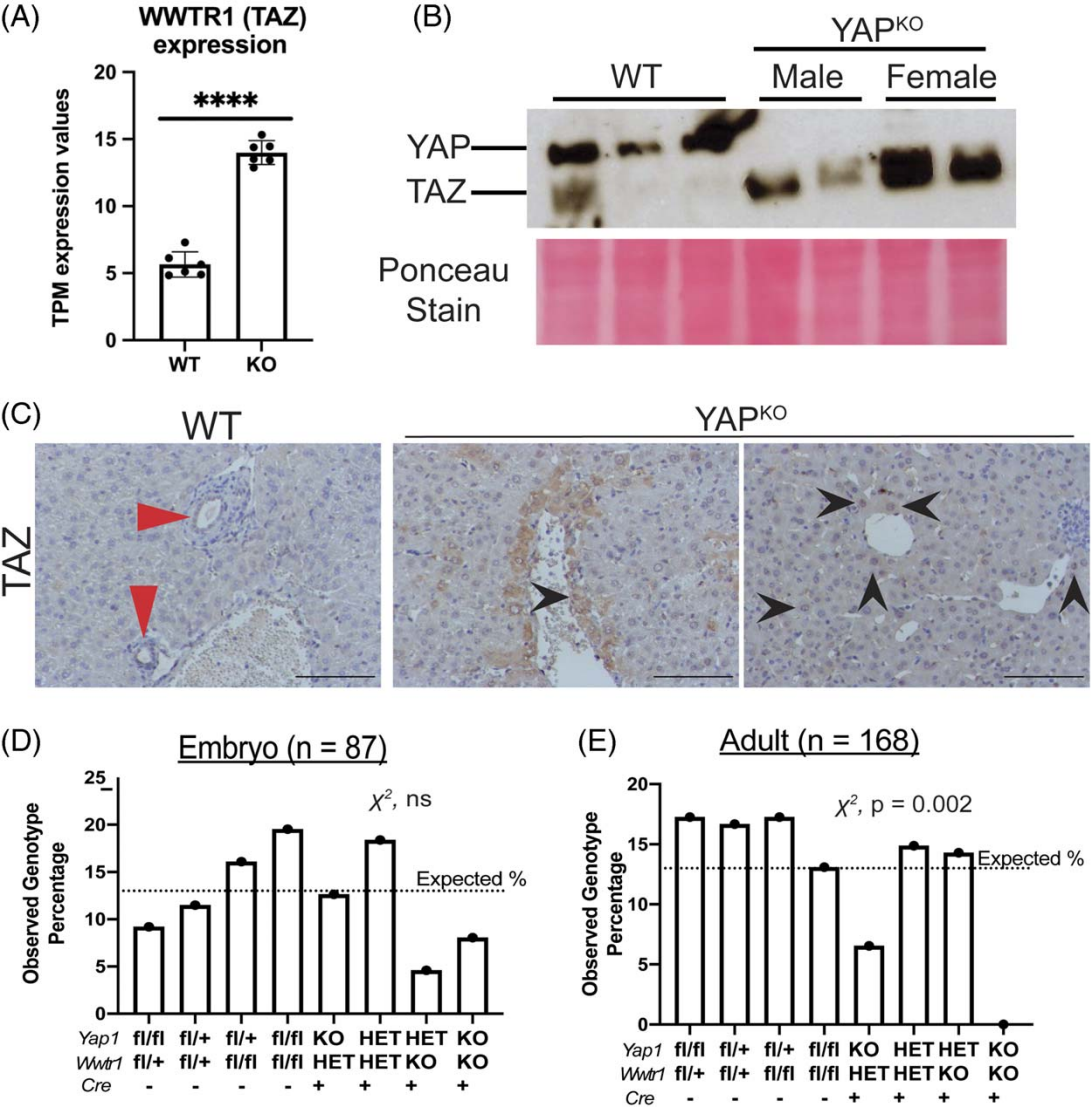
After YAP loss, TAZ is upregulated in hepatocytes, and YAP/TAZ double deletion leads to late embryonic demise. (A) TPM values comparing mRNA expression of *Wwtr1* in WT and YAP^KO^. (B) Western blotting for YAP and TAZ in YAP^KO^ versus WT mice. (C) IHC for TAZ in WT and KO mice; red arrows show positive bile duct staining of TAZ in WT, and black arrows highlight nuclear TAZ in hepatocytes. Scale bars 100 mm. (D and E) Genotype frequencies of the offspring from crossing (*Cre*^*+/-*^ *, Yap1*^*fl/+*^ *, Wwtr1*^*fl/+*^) × (*Cre*^*-/-*^ *, Yap1*^*fl/fl*^ *, Wwtr1*^*fl/fl*^), counting (D) embryos from E14-17 and (E) adult mice. Dashed lines reflect the expected frequency of 1/8 for all genotypes. Abbreviations: TAZ, transcriptional coactivator with PDZ-binding motif; TMP, transcript per million; WT, wild type; YAP, yes-associated protein 1.

We questioned how YAP^KO^ livers adapted to the lack of bile ducts. We noticed increased expression of transcriptional coactivator with PDZ-binding motif (TAZ), encoded by the gene *Wwtr1* (Figure 1A–C). TAZ also partners with TEAD transcription factors among others.^1,4^ YAP and TAZ are often studied in tandem due to their similarities in structure and function, and the potential for each to compensate for the other in regulating cell proliferation, survival, and stemness.^5^ However, investigations in various organs have identified functions unique to either YAP or TAZ in which the other could not compensate.^1,2,4^

In this study, we interrogated the role of TAZ in the absence of YAP in the early liver and foregut development using *Foxa3*-Cre to inactivate either one or both alleles of *Yap1* and *Wwtr1*. We present results showing the effects of deletion of both YAP and TAZ in early development and the impact of TAZ partial loss on the ability of the liver to adapt to chronic cholestasis in YAP^KO^ mice. Our results point to the unique roles of TAZ in injury repair independent of YAP.

## METHODS

### Animal models

C57BL/6 *Yap1*^fl/fl^ mice (Jackson Labs Stock No. 027929)^6^ were bred into C57BL/6 *ROSA*-stop^fl/fl^-EYFP mice. These mice were bred into C57BL/6 *Foxa3*-Cre mice described^7^ to create *Foxa3*-Cre *Yap1*^fl/fl^ ROSA-stop^fl/fl^-EYFP mice (YAP^KO^). Separately, FVB *Wwtr1*^fl/fl^ *Yap*1^fl/fl^ mice (Jackson Labs Stock No. 030532)^8^ were bred into C57BL/6 *Foxa3*-Cre mice to create *Foxa3*-Cre *Wwtr1*^fl/fl^ *Yap*1^fl/fl^ mice. Experimental animals are all F3 generation offspring of backcrosses with the original floxed mice. All animal studies were performed in accordance with the guidelines of the Institutional Animal Use and Care Committee (protocol number 22112055) at the University of Pittsburgh School of Medicine and the National Institutes of Health. All animals were group housed in ventilated cages under 12-hour light/dark cycles with access to enrichment, water, and standard chow diet *ad libitum.* Both male and female mice were used throughout the study, and littermates were used as wild-type (WT) controls. Mice were analyzed at E17.5, P1-2, P21, and 3–4 months of age. Analysis of serum liver function tests was performed by the clinical laboratories at the University of Pittsburgh Medical Center.

### Immunostaining

Adult and fetal livers were fixed in 10% formalin for 24–48 hours and stored in 70% ethanol, and paraffin-embedded 4 μm paraffin sections were cut, deparaffinized, and rehydrated. For immunohistochemistry (IHC), sections underwent antigen retrieval by the methods as described in Supplemental Table S1, http://links.lww.com/HC9/A418 for each primary antibody. The IHC protocol was performed as described.^3^ Hematoxylin and eosin staining was performed as described.^3^ For terminal deoxynucleotidyl transferase dUTP nick end labeling (TUNEL) staining, slides were treated with proteinase K (Millipore Cat. No. 21627) for 15 minutes at room temperature for antigen retrieval, and a TUNEL staining kit (Millipore Sigma, S7100) was used. Images were taken on a Zeiss Axioskop 40 inverted brightfield microscope. Whole slides were scanned at ×40 magnification using an Aperio AT2 slide scanner (Leica Biosystems). Cell and nuclei quantification was performed using Fiji/ImageJ.^9^

### Immunoprecipitation and western blotting

Whole cell protein lysate preparation was performed using radioimmunoprecipitation assay buffer. Immunoprecipitation was performed as described^10^ using 1 mg of precleared protein and A/G agarose beads (Santa Cruz, sc-2003), with ~2 mg of antibody targeting pan-TEAD (Cell Signaling Technology CS13295S, Rabbit). At least 30 μg of protein was used per sample for western blots, performed as described.^10,11^ We used primary antibody target YAP/TAZ (Cell Signaling Technology CS8418S Rabbit) at 1:500 dilution and horseradish peroxidase-conjugated secondary antibody at 1:5000 dilution (Mouse anti-Rabbit Light Chain Only HRP-conjugated, Cell Signaling Technology CS93702).

### Bile acid species detection and quantification

Bile acid profiling was performed as described.^3,12^ Livers were homogenized in water (100 mg tissue in 500 μL water), and then, 300 μL of methanol:acetonitrile (v/v, 1:1) was added to a 100 μL aliquot of liver homogenate. All the mixtures were vortexed for 2 minutes and centrifuged at 15,000 rpm for 10 minutes. Two microliters of the supernatants from all samples were injected into the ultraperformance liquid chromatography coupled with an SYNAPT G2-S quadrupole time-of-flight mass spectrometry (QTOFMS, Waters Corporation, Milford, MA). The column type is Acquity UPLC BEH C18 column (2.1 × 100 mm, 1.7 μm). Protocols for the use of a mobile phase gradient and QTOFMS system were reported.^12,13^ Bile acid species were quantified by measuring their relative abundance as the AUC for each species using standards for comparison.

Serum bile acid quantification was done using the Mouse Total Bile Acids Assay Kit (CrystalChem #80471). Serum samples were diluted 1:5 and were analyzed per kit instructions.

### RNA extraction and RNA-sequencing analysis

RNA was extracted from frozen whole liver tissue using QIAGEN Rneasy Mini Kit (Cat. 74104). DNA digestion and removal were performed using Rnase-free Dnase Set (QIAGEN Cat. 79254). Purified, high-quality RNA from 3 female YAP^KO^ mice with 3 female WT littermates (C57Bl6) and 3 female YAP^KO^ TAZ^HET^ mice with 3 female WT littermates (mixed background) were sent to Novogene Co. (Sacramento, CA) for cDNA library preparation and RNA sequencing by Illumina Novaseq 6000 using paired-end 150bp reads, with 20 million reads per end per sample. RNA-sequencing data generated from this study are available at Gene Expression Omnibus (GEO), Series GSE213815.

Raw sequencing data were processed using CLC Genomics Workbench 20.0.3 (QIAGEN) for quality control and aligned to the *Mus musculus* genome version GRCm38.p6. Reads assigned to each gene underwent trimmed mean of M-values normalization, and differential expression analysis was performed using *edgeR* ^14^ within CLC Genomics to compare YAP^KO^ versus WT littermates and YAP^KO^ TAZ^HET^ versus WT littermates. The top differentially expressed genes were filtered by adjusted *p*-value q < 0.05 and fold change greater than 2 for subsequent downstream pathway analysis using Ingenuity Pathway Analysis (IPA, QIAGEN), Gene Set Enrichment Analysis (GSEA), Molecular Signature Database (MsigDB),^15^ and Enrichr.^16,17^

### Public data mining of ChIP-Seq data to identify TEAD targets

Two public ChIP-Seq data sets were mined in this study to identify downstream genes potentially being regulated by TEAD transcription factors. The first was downloaded from ENCODE database^18,19^ for ChIP-Seq in HepG2 cells; peaks for TEAD1, TEAD3, and TEAD4 binding sites were called by the ENCODE website pipeline with default parameter settings (accession IDs ENCSR497JLX, ENCSR666QNP, and ENCSR000BRP). The second was downloaded from GEO (GSE107860).^20^ Raw mouse ChIP-sequencing data were collected for TEAD4. Raw reads were trimmed by Trimmomatic^21^ and aligned to mouse reference genome mm10 by the Burrows-Wheeler aligner.^22^ Peak calling was performed by tool MACS2^23^ comparing input sample and immune-precipitation samples. For all 3 studies, peak regions were annotated to genes by R/Bioconductor packages ChIPpeakAnno^24,25^ and ChIPseeker.^26^ Genes involved in peaks with *p*-value ≤1E-7 were selected as the TEAD target genes for downstream analysis. For our final list of potential TEAD targets, we selected 3773 genes that were present in all 4 data sets as potentially targeted by TEAD genome binding.

### Quantification and statistical analysis

Data are presented as mean ± SD using GraphPad PRISM version 9.1.2 software. *p* < 0.05 was considered statistically significant, except for individual bile acid species comparisons for which a false discovery rate of 0.1 was used (*p < 0.05, **p < 0.01, ***p < 0.001, and ****p < 0.0001). Data were analyzed using a 2-tailed unpaired Student *t* test or Mann-Whitney test where 2 groups were being compared. In cases where more than 2 groups were being compared, 1-way ANOVA or Kruskal-Wallis tests were used with Sidak test to correct for multiple comparisons. A Pearson correlation coefficient was determined to assess the relationship between 2 continuous variables.

## RESULTS

### Loss of 1 or both copies of *Wwtr1* in addition to *Yap1* results in selective embryonic lethality

We found that *Wwtr1* expression (the gene encoding TAZ) was significantly upregulated in YAP^KO^ mice (Figure 1A), and the TAZ protein level was significantly increased in YAP^KO^ mice (Figure 1B). Using IHC, we show that, at the baseline, TAZ expression can be seen in the bile ducts but not in hepatocytes (Figure 1C, red arrows). In contrast, YAP^KO^ mice show increased levels of cytoplasmic and nuclear TAZ in hepatocytes, especially in the portal region (Figure 1C, black arrows).

We next combined YAP^fl/fl^ and TAZ^fl/fl^ mice (FVB background) with *Foxa3*-Cre mice (C57BL/6 background), with a final breeding cross of [*Cre*^*+/−*^, *Yap1*^*fl/+*^ *, Wwtr1*^*fl/+*^] x [*Cre*^*−/−*^ *, Yap1*^*fl/fl*^ *, Wwtr1*^*fl/fl*^], which resulted in a variety of combinations of allele disruption in early foregut endoderm development. The observed variability among embryo genotype ratios may be due to the small sample size and a large number of possible genotypes, and it does not represent a statistically significant difference by the chi-squared test. However, we observed significant disparities in the expected Mendelian ratios of genotypes among live births (Figure 1D, E). YAP/TAZ double knockout (DKO) mice were never found among live births but were present at expected ratios among embryos harvested between E14 and E17 (Figure 1D, E). We also observed a 50% decrease in the expected birth rate of mice that have lost both *Yap1* alleles but only 1 allele of *Wwtr1* (YAP^KO^ TAZ^HET^). Further examination revealed that surviving YAP^KO^ TAZ^HET^ mice were almost exclusively female, while males died around the time of birth.

**FIGURE 2:**
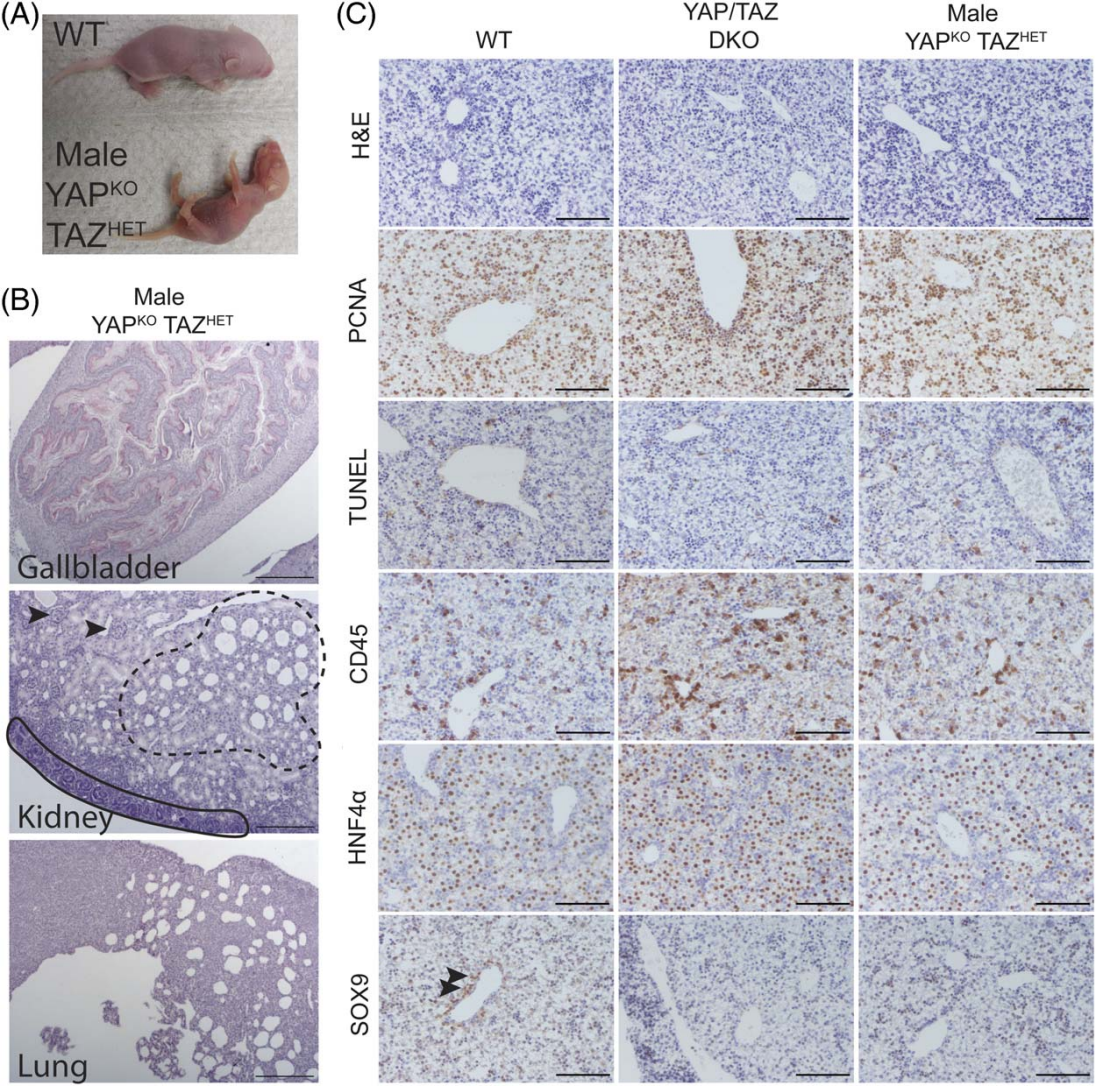
YAP and TAZ deletion in early embryonic development leads to embryonic lethality but does not affect gross liver development although both YAP^KO^ TAZ^HET^ and YAP/TAZ DKO livers show impaired bile duct formation. (A) Gross image of a WT pup at postnatal day 1 and littermate pup, a male YAP^KO^ TAZ^HET^ mouse, which died shortly after, showing its thin skin, underdeveloped ears, and prominent blood vessels throughout. (B) H&E staining of the gallbladder (scale bar 200 mm), lungs (scale bar 200 mm), and kidneys (scale bar 100 mm) of the same pup shown in A. Arrows point to glomeruli; solid black line shows a layer of S-shaped bodies, and dashed black line shows dilation of renal tubules. (C) H&E stain and IHC for PCNA, TUNEL, CD45, HNF4a, and SOX9 comparing WT, YAP/TAZ DKO, and YAP^KO^ TAZ^HET^ male mice at E17-18 (scale bar 100 mm). Arrows point to developing bile ducts. Abbreviations: DKO, double knockout; H&E, hematoxylin and eosin; PCNA, proliferating cell nuclear antigen; HET, heterozygote; KO, knockout; TAZ, transcriptional coactivator with PDZ-binding motif; TUNEL, terminal deoxynucleotidyl transferase dUTP nick end labeling; WT, wild type; YAP, yes-associated protein 1.

One of the few male YAP^KO^ TAZ^HET^ pups that we identified died at postnatal day 1, exhibiting severe sickness, including jaundice and pale skin with prominent vessels resembling earlier-stage embryos (Figure 2A). Several developmental defects were revealed by histological analysis (Figure 2B). For example, the gallbladder was filled with keratinized papillary growths. The kidneys showed severe abnormalities: many S-shaped bodies were still visible (Figure 2B, solid line), and we found that few developed glomeruli (Figure 2B arrows), along with significant tubular dilatation suggesting cystic disease (Figure 2B, dashed line). The lungs were severely underdeveloped and had insufficient airspaces, which could explain why the pup survived until birth but then quickly decompensated.

To determine whether disruption of *Wwtr1* also had an impact on early liver development in the setting of *Yap1* loss, embryonic livers (E17-18) from DKO mice and male YAP^KO^ TAZ^HET^ mice were analyzed histologically (Figure 2C). Liver morphology and hepatocyte appearance were not significantly altered in either model, as shown by hematoxylin and eosin and HNF4a staining (Figure 2C). Staining for SOX9 highlights the formation of bile ducts in WT but not in DKO or in male YAP^KO^ TAZ^HET^ mice; this phenotype is similar to what we previously observed in YAP^KO^ mice.^3^ Neither model showed signs of increased apoptosis (TUNEL stain) or differences in proliferation rates (proliferating cell nuclear antigen). However, we observed differences in the distribution of immune cells (CD45), with greater numbers appearing near the portal veins of DKO and male YAP^KO^ TAZ^HET^ mice; this may be related to the absence of functional bile ducts, which would normally be in this region and whose signaling may influence hematopoiesis and inflammatory cell distribution. The embryonic livers of DKO and male YAP^KO^ TAZ^HET^ mice were, thus, not severely injured at E17-18, suggesting an extrahepatic source of lethality in both models.

### *Foxa3*-Cre YAP^KO^ TAZ^HET^ females survive long-term showing signs of adaptation to bile duct loss

**FIGURE 3:**
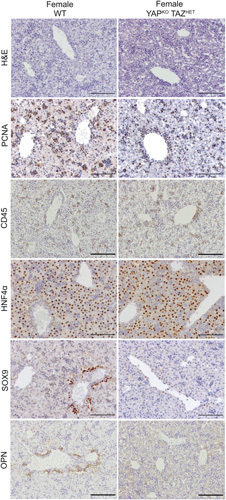
YAP^KO^ TAZ^HET^ female livers at postnatal days 1 and 2 are overall similar to female WT livers, except for failure of bile duct formation similar to YAP^KO^ livers, as shown by H&E staining and IHC for PCNA, CD45, HNF4α, SOX9, and OPN (scale bar 100 mm). Abbreviations: HET, heterozygote; HNF4α, hepatocyte nuclear factor 4 alpha; KO, knockout; OPN, osteopontin; PCNA, proliferating cell nuclear antigen; SOX9, SRY-box transcription factor 9; TAZ, transcriptional coactivator with PDZ-binding motif; WT, wild type; YAP, yes-associated protein 1.

Next, we turned our attention to the female YAP^KO^ TAZ^HET^ mice, which survived into adulthood, unlike their male genotypic counterparts. Analysis of these mice at postnatal days 1 and 2 revealed a histological phenotype grossly similar to female WT (Figure 3), with comparable levels of cell proliferation (proliferating cell nuclear antigen) and distribution and morphology of hepatocytes (HNF4a). There was a small increase in the number of CD45-positive inflammatory cells around the portal veins although less pronounced than in male YAP^KO^ TAZ^HET^ mice (Figures 2C and 3). Finally, we observed impairments in bile duct formation similar to YAP^KO^ mice, with the presence of individual SOX9-positive cells around the portal vein but no functional tubular structures^3^ (Figure 3).

**FIGURE 4:**
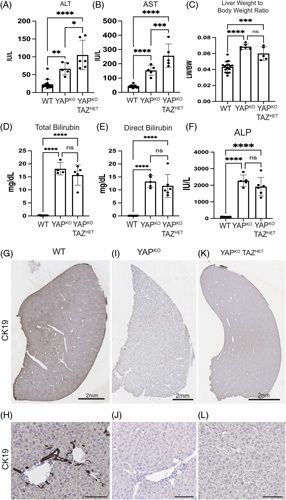
Serum biochemistry shows worsened hepatocellular injury in adult female YAP^KO^ TAZ^HET^ mice compared with YAP^KO^. Serum levels of (A) alanine aminotransferase (ALT), (B) aspartate aminotransferase (AST), (D) total bilirubin, (E) direct bilirubin, and (F) alkaline phosphatase in WT, YAP^KO^, and YAP^KO^ TAZ^HET^ mice at 3–4 months old. (C) Liver-to-body weight ratio of YAP^KO^ and YAP^KO^ TAZ^HET^ mice relative to WT mice. (G)–(L) Slide scans of adult liver lobes and insets of portal vein areas of (G and H) WT, (I and J) YAP^KO^, and (K and L) female YAP^KO^ TAZ^HET^ mice, all stained for CK19 to highlight the bile ducts, which are not present in I-L. Scale bars G, I, and K are 2 mm, and H, J, and L are 100 mm. Abbreviations: ALP, alkaline phosphatase; ALT, aminotransferase; AST, aspartate aminotransferase; CK19, cytokeratin-19; WT, wild type; YAP, yes-associated protein 1.

We next examined the liver function of these mice at 3–4 months of age (similar to YAP^KO^ mice that we analyzed previously).^3^ Adult YAP^KO^ TAZ^HET^ mice had similar liver-to-body weight ratio as YAP^KO^ mice, but they had increased levels of alanine aminotransferase (ALT) and aspartate aminotransferase (AST), suggesting increased hepatocellular injury (Figure 4A–C). YAP^KO^ TAZ^HET^ mice also had extremely high levels of total and direct bilirubin and alkaline phosphatase (ALP) (Figure 4D–F). Histologically, they resembled adult YAP^KO^ mice and exhibited an absence of intrahepatic ducts, with no evidence of functional biliary regeneration, as shown by IHC staining of CK19 (Figure 4 G–L).

**FIGURE 5:**
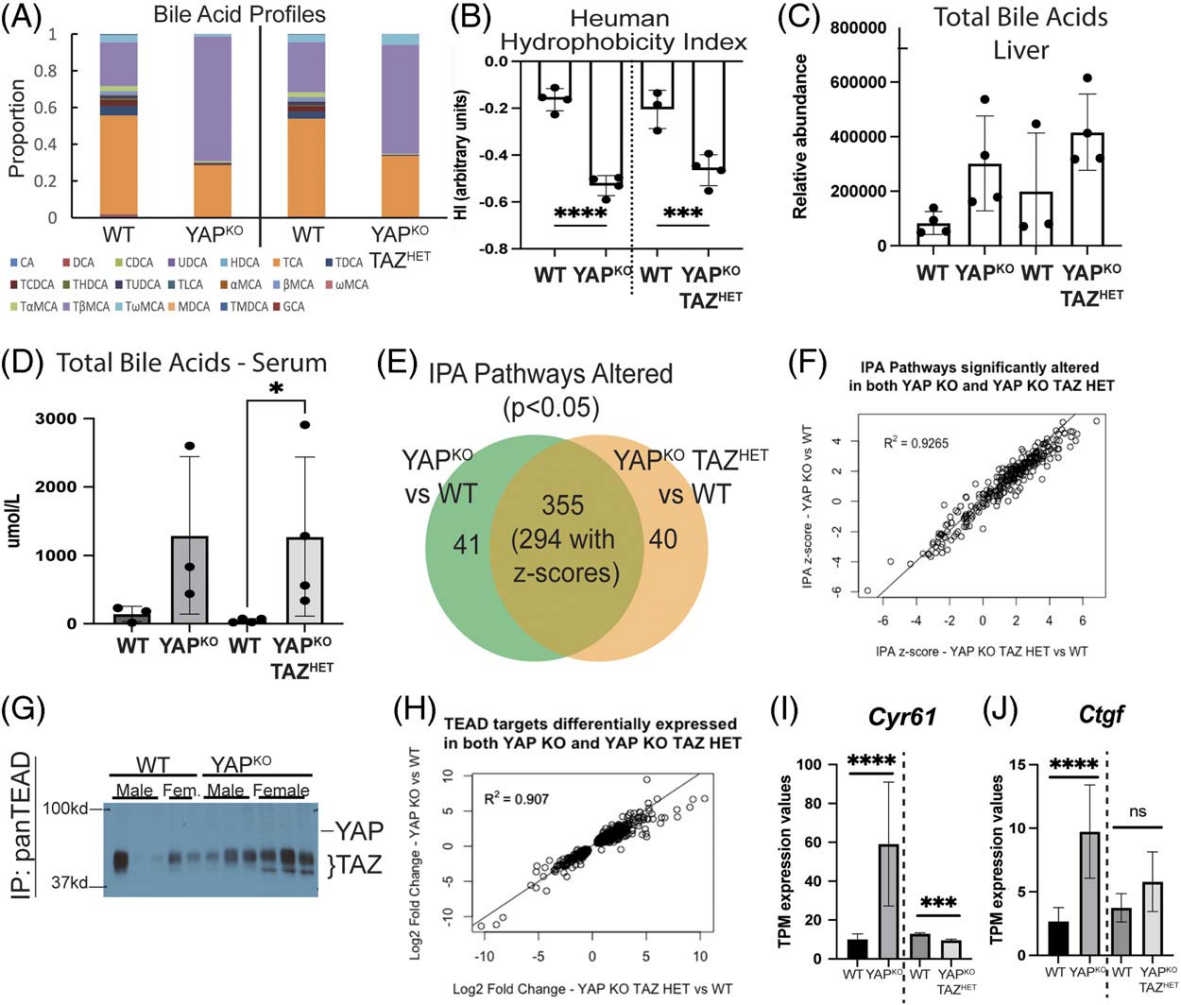
Bile acid profiling and RNA-sequencing analysis show phenotypic concordance between adult YAP^KO^ and YAP^KO^ TAZ^HET^ females, except for a subset of canonical YAP-TAZ target genes. (A) Bile acid profiling from frozen liver tissue, along with (B) Heuman hydrophobicity index of liver bile acids and (C) total bile acids, comparing YAP^KO^ and YAP^KO^ TAZ^HET^ adult females with corresponding WT controls. (D) Concentration of total bile acids in the serum of YAP^KO^ and YAP^KO^ TAZ^HET^ adult females with corresponding WT controls. (E) Venn diagram showing the overlap in altered IPA pathways based on differential gene expression data comparing YAP^KO^ and YAP^KO^ TAZ^HET^ to corresponding littermate WT controls. (F) Scatterplot of IPA *z*-scores indicating the suspected upregulation or downregulation of each altered pathway. (G) Western blot of YAP and TAZ following immunoprecipitation with a pan-TEAD antibody. (H) Scatterplot comparing differential expression of TEAD targets in YAP^KO^ versus WT and YAP^KO^ TAZ^HET^ versus WT. (I and J) TPM expression values of *Cyr61* and *Ctgf* in either YAP^KO^ versus WT or YAP^KO^ TAZ^HET^ versus WT. Abbreviations: bMCA, beta-muricolic acid; CA, cholic acid; CDCA, chenodeoxycholic acid; DCA, deoxycholic acid; GCA, glycocholic acid; HDCA, hyodeoxycholic acid; IPA, ingenuity pathway analysis; MDCA, murideoxycholic acid; TaMCA, tauro-alpha-muricholic acid; TbMCA, tauro-beta-muricholic acid; TCA, taurocholic acid; TCDCA, taurochenodeoxycholic acid; TDCA, taurodeoxycholic acid; THDCA, taurohyodeoxycholic acid; TLCA, aMCA alpha-muricolic acid; TMDCA, tauromurideoxycholic acid; TUDCA, tauroursodeoxycholic acid; TwMCA, tauro-omega-muricholic acid; UDCA, ursodeoxycholic acid; wMCA, omega-muricolic acid; YAP, yes-associated protein 1.

Based on these data, we aimed to identify what could be causing increased hepatocellular damage in YAP^KO^ TAZ^HET^ mice. Since changes in bile acid composition were a major component of the adaptations of YAP^KO^ mice,^3^ we decided to analyze the bile acid profiles of adult YAP^KO^ TAZ^HET^ livers as well. We found that both models had very similar changes in their bile acid pools compared with their WT littermates (Figure 5A), with taurobetamurocholic acid being the most abundant species by far in both disease models. The overall hydrophobicity of the liver bile acid pool was significantly decreased in both models with no significant change between them (Figure 5B). There was a general reduction in bile acid diversity although the high variability in total bile acid quantities across samples and the large number of comparisons meant that none reached statistical significance (Figure 5C). We also saw a dramatic increase in the quantity of bile acids in the serum of YAP^KO^ TAZ^HET^ mice compared with WT (Figure 5 D), just as in the YAP^KO^ mice.

### In the absence of YAP, TAZ regulates a subset of TEAD targets, which influences cell cycling and macrophage-mediated inflammation

We next performed RNA-sequencing analysis comparing female YAP^KO^ mice to littermate controls (C57Bl6 background) and female YAP^KO^ TAZ^HET^ mice to littermate controls (mixed FVB/C57Bl6 background). We found that 355 IPA signaling pathways significantly altered in both YAP^KO^ and YAP^KO^ TAZ^HET^ mice, most of which had highly concordant *z*-scores (Figure 5E, F). These altered pathways are very similar to those we described in our previous study of YAP^KO^ mice.^3^ We observe the upregulation of regenerative pathways favoring cell proliferation and survival, alongside a downregulation of metabolic pathways, including fatty acid metabolism, bile acid metabolism, and oxidative metabolism. The overall similarity of the altered pathways and the phenotype in YAP^KO^ and YAP^KO^ TAZ^HET^ mice suggests that TAZ heterozygosity did not affect the global genetic adaptations in these mice.

We next looked for more subtle changes, hypothesizing that TAZ heterozygosity may have altered the expression of genes that are targets of TEAD transcription factors, given the close relationship between TAZ and TEAD. Indeed, immunoprecipitation for TEAD transcription factors (using a pan-TEAD antibody) revealed much higher levels of TAZ bound to TEAD factors in YAP^KO^ mice in comparison to WT mice (Figure 5G). We compared the differentially expressed genes in each mouse model to publicly available ChIP-Seq datasets mapping TEAD binding sites throughout the genome in either the mouse liver or the HepG2 cell line.^18–20^ Out of 3773 potential TEAD targets identified in all 4 data sets, we found that about 26% were altered in YAP^KO^ mice, and 34% were altered in YAP^KO^ TAZ^HET^ mice relative to WT. Nine hundred sixty-eight targets were altered in both models, covering many major injury response pathways (Supplemental Figure S1A, http://links.lww.com/HC9/A418). The log2 fold change of these genes was impressively concordant across both models, with *R*^2^ of 0.907 (Figure 5H). These genes were mostly not affected by TAZ heterozygosity and, thus, may be regulated by other signaling pathways that are responding to cholestatic liver injury, as has been described before.

Next, we focused on the targets altered in 1 but not both mouse models. Curiously, we found that *Cyr61*, a well-known target of YAP and TAZ, was significantly upregulated in YAP^KO^ mice relative to WT but was significantly decreased after the loss of 1 copy of TAZ (Figure 5I). This suggests that the expression of *Cyr61* in response to injury is directly regulated by TAZ in YAP^KO^ mice. *Ctgf*, another YAP/TAZ target, was similarly upregulated in YAP^KO^ mice but was unchanged relative to WT after the loss of 1 copy of TAZ (Figure 5J). We found 298 genes whose expression followed a similar pattern of upregulation in YAP^KO^ mice (fold change >2 and q-value <0.05) but were unaltered or downregulated in YAP^KO^ TAZ^HET^ mice (either q-value >0.05 or fold change < 0 and q < 0.05) (Supplemental Figure S1B, http://links.lww.com/HC9/A418, and Supplemental Table S2, http://links.lww.com/HC9/A418). Sixty-six of these genes were also potential TEAD targets. These genes were enriched in GO terms and pathways associated with G1/S cell cycle transitions, E2F targets, mitosis, and regulation of apoptosis, reflective of a more targeted program often associated with oncogenic YAP/TAZ signaling^27,28^ (Supplemental Figure S1A and C, http://links.lww.com/HC9/A418). These genes were also enriched in regulators of cytokine production and monocyte recruitment (Supplemental Figure S1C, http://links.lww.com/HC9/A418). This may reflect a specific program of genes particularly sensitive to TAZ/TEAD regulation in the absence of YAP.

**FIGURE 6:**
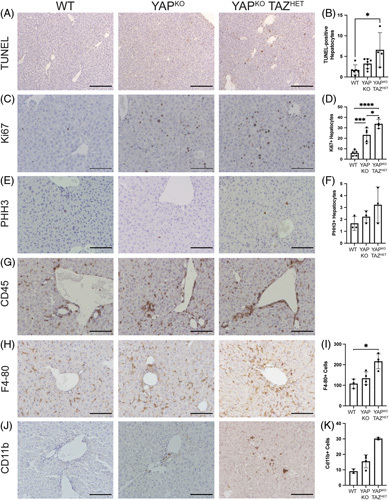
YAP^KO^ TAZ^HET^ mice have increased cell cycling, cell death, and total macrophage numbers with increased monocyte recruitment. IHC for (A) TUNEL, (C) Ki67, (E) PHH3, (G) CD45, (H) F4-80, and (J) CD11b, with respective quantifications in (B) TUNEL-positive hepatocytes, (D) Ki67-positive hepatocytes, (F) PHH3-positive hepatocytes, (I) number of F4-80-positive cells, and (K) number of CD11b positive cells. Scale bars: (A) 200 mm; (C), (E), (G), (H), and (J) 100 mm. Data were analyzed by Kruskal-Wallis (TUNEL, PHH3, F4/80) or 1-way ANOVA (Ki67) with the Sidak multiple comparison tests. Abbreviations: CD11b, integrin αM; CD45, leukocyte common antigen; PHH3, phosphohistone H3; TUNEL, terminal deoxynucleotidyl transferase dUTP nick end labeling; WT, wild type; YAP, yes-associated protein 1.

We then asked whether these gene expression changes had a functional impact on YAP^KO^ TAZ^HET^ livers. We assessed cell death through TUNEL staining (Figure 6A, B) and show that it was significantly increased in YAP^KO^ TAZ^HET^ hepatocytes compared with WT and trended toward an increase compared with YAP^KO^ although the overall level of hepatocyte death was low across all samples, reflecting their successful adaptation. There was an increase in Ki67-positive hepatocytes in YAP^KO^ mice, which was further surpassed in YAP^KO^ TAZ^HET^ mice, suggesting increased cell cycling (Figure 6C, D). However, mitotic activity was not significantly altered, as shown by the phosphohistone H3 (PHH3) staining, which was very low across most samples (Figure 6E, F). Finally, given the gene expression changes pointing to alterations in cytokine signaling, we looked at the inflammatory cell population, which was generally increased in both YAP^KO^ and YAP^KO^ TAZ^HET^ livers (CD45 staining, Figure 6G). The overall quantity of macrophages, evidenced by F4-80 staining, was significantly increased in YAP^KO^ TAZ^HET^ livers compared with both WT and YAP^KO^ (Figure 6H, I). We also found an increase in CD11b+ cells in YAP^KO^ TAZ^HET^ livers, suggesting increased monocyte recruitment (Figure 6J, K).

## DISCUSSION

Our data shed light on the relationship between YAP and its paralog TAZ in liver development and chronic cholestatic injury. Although some studies have shown that TAZ plays a role in biliary development, TAZ is unable to compensate for the loss of YAP to restore bile duct morphogenesis.^3,29,30^ We consistently observed impaired bile duct development throughout the mouse models in which YAP was deleted, regardless of TAZ status. We also observed a subtle increase in the abundance of immune cells around the portal veins in embryonic livers of all disease models that we present here, which may be related to their disrupted bile duct formation. However, complete loss of both YAP and TAZ does not grossly impair the development of the hepatic parenchyma.

Interestingly, loss of both YAP and TAZ from the foregut endoderm resulted in embryonic lethality around E17-18. This result directly contrasts with the study of *Alb*-Cre YAP/TAZ DKO mice, which were described by Verboven et al.^31^ *Alb*-Cre is specific to the liver only and completes recombination in late development, yet these mice were not embryonic lethal, instead reaching adulthood with moderate bile duct paucity. This further supports that YAP and TAZ losses do not impair hepatocyte development and reveal a distinct, essential function for both YAP and TAZ in the early development of the foregut endoderm outside of the liver. However, we had insufficient tissue to investigate the specific organ functions disrupted in this case beyond a cursory overview. The 1 pup that we identified as a male YAP^KO^ TAZ^HET^ showed significant organ dysfunction. While our Cre-line is known to affect foregut endoderm derivatives, we have evidence suggesting that it also affects developing kidneys, which originates from mesoderm (data not shown), and it is known that TAZ loss in development leads to significant cystic renal disease.^32^ Renal dysfunction could cause poor urine output, leading to insufficient amniotic fluid and subsequently poor lung development, a well-described constellation of findings known as Potter syndrome.^33,34^ Thus, impaired lung development may not be directly related to a genetic defect in this pup.

A similar study in zebrafish revealed developmental abnormalities in *yap*^*−/−*^ *taz*^*−/−*^ and *yap*^*−/−*^ *taz*^*+/−*^ offspring.^30^ Complete loss of YAP and TAZ in zebrafish embryos (whole-body deletion) caused dramatic shortening of the posterior body, while preservation of 1 copy of *taz* resulted in malformations of the digestive system specifically.^30,35^ The *yap*^*−/−*^ *taz*^*+/−*^ zebrafish exhibited the absence of gut rotation and the presence of bilateral liver and pancreas, as well as significantly decreased proliferation of liver progenitor cells. While YAP^KO^ TAZ^HET^ male mouse embryos exhibited digestive tract developmental defects, we did not observe the presence of multiple small liver buds or defects in liver progenitor proliferation in the mouse in either sex. There may be additional compensatory mechanisms regulating liver development in the mouse, which are absent in zebrafish.

There are no reports of similarly dramatic sex dimorphism in phenotypes resulting from alteration in YAP or TAZ activity although mild sex differences have been seen in endothelial cell function.^36^ Whole-body loss of TAZ in mice has been shown to cause abnormalities evident shortly after birth such as increased air-space diameter in the lungs and polycystic kidney disease, suggesting unique roles in the development of these organs.^32,37^ Another important variable may be the genetic background of the mice: unknown combinations of small nucleotide polymorphisms caused by mixed backgrounds could also affect the phenotypic outcome. Our breeding scheme significantly reduced variability due to mixed backgrounds since we consistently used F3 generation animals as our experimental subjects, providing consistency across litters. Our data show that YAP is critical for bile duct formation regardless of mouse strain and background, which strengthens the applicability of our data to other disease models. Furthermore, the wild-type mice of mixed background did not show significant differences compared to the C57Bl6 wild-type mice. All of our model comparisons used litter mate controls to account for the effect of background differences, and the global similarity between the adult female YAP^KO^ and YAP^KO^ TAZ^HET^ suggests that the background of the mice did not impact the overall phenotype.

We also show that TAZ heterozygosity in adult female YAP^KO^ mice mildly worsened their phenotype, and TAZ seems to regulate a small subset of genes affecting cell proliferation and monocyte recruitment to the injured liver. In adult YAP^KO^ mice, TAZ may partner with TEAD to regulate a subset of genes related to mitosis, cell proliferation, inflammation, and apoptosis. The majority of putative TEAD targets were unaffected by TAZ heterozygosity in the context of YAP loss, suggesting that they were regulated by other genetic circuits or are completely YAP-dependent. The overall metabolic reprogramming in YAP^KO^ mice^3^ was mostly unaffected by TAZ heterozygosity, as shown by the dramatic similarity across altered IPA pathways and putative TEAD targets in both adult female YAP^KO^ and YAP^KO^ TAZ^HET^. The pattern of gene expression changes and altered pathways (Figure 5 and Supplemental Figure S1A, http://links.lww.com/HC9/A418) matches our previous description of YAP^KO^ mice^3^ and reflects a global reprogramming of hepatocytes promoting survival and regeneration while altering bile acid metabolism to reduce hepatotoxicity.

However, certain gene targets may be especially sensitive to the presence and quantity of TAZ and may require a certain threshold of TAZ activity to promote expression, independent of YAP. The loss of *Cyr61* and *Ctgf* upregulation following TAZ heterozygosity in YAP^KO^ livers suggests that these 2 genes are extremely responsive to TAZ/TEAD regulation in hepatocytes. It has been shown that TAZ, but not YAP, can homodimerize and interact with TEAD alone to regulate gene expression, and TAZ and/or YAP binding to TEAD switches its function from repressing to promoting target gene expression.^4^ TAZ is also known to bind to other transcription factors, which may be playing a role in regulating non-TEAD targets. Our results suggest that these select targets impact cell death and cell cycling, and perhaps restrict the completion of mitosis, resulting in the observed increase in Ki67-positivity but preventing a significant change in proliferation overall (PHH3). These targets also impacted hepatocyte crosstalk with macrophages but paradoxically resulted in increased macrophage recruitment. Although other studies have shown that macrophage recruitment is diminished following YAP and TAZ loss, these differences may be explained by the absence of cholangiocytes and minimal ductular reaction in our model, which would normally be major contributors to inflammatory signaling in the setting of injury.^31,38^

On the one hand, TAZ in the absence of YAP could be tightly managing the injury repair response. Several studies have shown that TAZ upregulation (without YAP alteration) promotes inflammation, macrophage recruitment, fibrogenesis, and worsened hepatocellular injury,^31,38–40^ while here we show that downregulation of TAZ leads to similar effects (but in the absence of YAP). This suggests that TAZ needs to be tightly regulated in healthy livers, and interactions between TAZ and YAP may critically alter their functions under injury conditions. On the other hand, TAZ may be promoting hepatocyte survival, as shown previously,^41^ and TAZ heterozygosity may lead to increased cell death, indirectly promoting inflammation and cell cycling.

These data point to the unique roles of TAZ in liver development and injury repair independent of YAP and reveal heretofore unknown relationships between YAP and TAZ. Further studies are needed to clarify the distinct functions of YAP and TAZ, and how they change when one or the other is absent or activated. This may significantly impact how we target them therapeutically in a wide range of liver pathologies.

## FUNDING INFORMATION

Funding was provided by NIH grants 2T32EB001026 and 1F30DK121393-01A1 to Laura M. Molina; 5R01CA204586-05, 1R01DK62277 and Endowed Chair for Experimental Pathology to Satdarshan P.S. Monga; NIH grant R01DK126875 to X.M.; and NIH grant 1P30DK120531 to Pittsburgh Liver Research Center for services provided by the Clinical Biospecimen Repository and Processing Core and the Genomics and Systems Biology Core. Funding was also provided to Adelya Gabdulkhakova by the Fulbright Program. This research was also supported in part by the University of Pittsburgh Center for Research Computing, RRID:SCR_022735, through resources provided including the HTC cluster (NIH S10OD028483).

## CONFLICTS OF INTEREST

The authors have no conflicts to report.

## Supplementary Material
