# Supplementary material for: Loss of TAZ after YAP deletion severely impairs foregut development and worsens cholestatic hepatocellular injury
Source: Hepatol Commun. 2023 Aug 9;7(9):e0220. doi: 10.1097/HC9.0000000000000220 (PMC10412434; doi:10.1097/HC9.0000000000000220)
Supplement: Supplementary file 1 [file hc9-7-e0220-s001.docx]

**Supplementary Information**

**Supplementary Figure 1.** Venn diagram showing overlap of potential TEAD targets differentially altered in YAP KO mice or YAP^KO^ TAZ^HET^ mice relative to their corresponding WT littermates, along with the MSIGDB pathways selectively enriched in each group of genes. B) Heatmap of 298 genes significantly upregulated in YAP KO mice but either unchanged or downregulated in YAP^KO^ TAZ^HET^ mice relative to WT (FC, fold change). C) Enrichr analysis of these 298 genes revealed significant enrichment of several GO Biological Process terms.


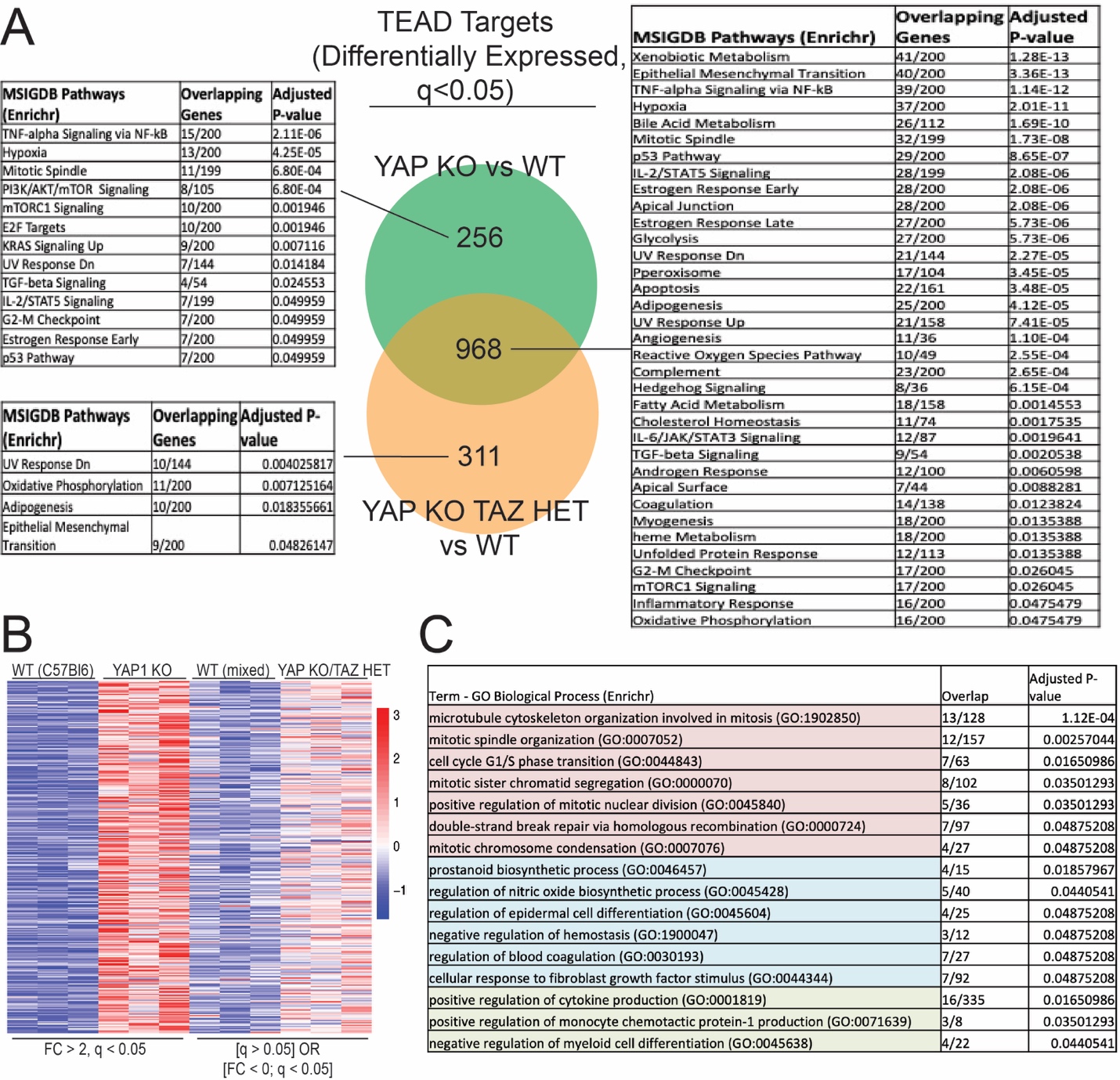


**Supplementary Table 1.** List of antibody names, catalog numbers, and protocol specifications for immunohistochemistry.

| Antibody Target | Antibody Species | Source | Identifier | Antigen Retrieval | Antibody Dilution |
| --- | --- | --- | --- | --- | --- |
| Primary Antibodies | | | | | |
| SOX9 | Rabbit | EMD Millipore | Cat#ab5535 | Pressure cooker, 20 minutes, sodium citrate buffer pH 6 | 1:2000 |
| HNF4a | Rabbit | Cell Signaling Technology | Cat#CS3113 |  | 1:100 |
| TAZ | Mouse | Abcam | CAT#ab242313 |  | 1:50 |
| Osteopontin (OPN) | Mouse | R&D Systems | Cat#AF808-SP |  | 1:100 |
| CK19 | Rat | Developmental Studies Hybridoma Bank (DSHB) | Cat#TROMAIII | Pressure cooker, 20 minutes, DAKO buffer (Agilent S1699) | 1:50 |
| Phospho-histone H3 (PHH3) | Rabbit | Cell Signaling Technology | Cat#9715s |  | 1:100 |
| CD45 | Rat | Santa Cruz Biotechnology | Cat#sc53665 |  | 1:100 |
| CD11b | Rat | Biolegend | Cat#101201 |  | 1:100 |
| F4-80 | Rat | Serotech | Cat#MCA497GA | Proteinase K solution, 6 minutes | 1:100 |
| PCNA | Mouse | Santa Cruz Biotechnology | Cat#sc-56 | Microwave, 60% power, 12 minutes, 1% zinc sulfate | 1:4000 |
| Ki67 | Rabbit | Sigma | Cat#NM-SP6 | Microwave, 60% power, 12 minutes, Tris EDTA buffer pH 9 | 1:100 |
| Secondary Antibodies | | | | | |
| IgG Donkey Anti-Rabbit, Biotin, Polyclonal | Donkey | Millipore Sigma | Cat#AP182B |  | 1:500 |
| IgG Goat Anti-Mouse, Biotin, Polyclonal | Goat | Millipore Sigma | Cat#AP181B |  | 1:500 |
| IgG Goat Anti-Rat, Biotin, Polyclonal | Goat | Millipore Sigma | Cat#AP183B |  | 1:500 |

**Supplementary Table 2.** List of genes significantly upregulated in YAP KO mice but either unchanged or downregulated in YAP^KO^ TAZ^HET^ mice relative to WT, and whether or not they are a potential TEAD target.

| Gene Name | Potential TEAD Target | Gene Name | Potential TEAD Target | Gene Name | Potential TEAD Target |
| --- | --- | --- | --- | --- | --- |
| Ptgs2 |  | Zbtb16 |  | Fbln7 | YES |
| Hesx1 |  | Nptx1 |  | Gpc3 | YES |
| Fcrls |  | Cenpi |  | Pla2g4a |  |
| Thbs4 |  | H1f4 |  | Trip13 |  |
| Omd |  | Cfap69 |  | Hells | YES |
| Tubb2b |  | Edn1 | YES | Sestd1 |  |
| Trpc2 |  | Nectin4 |  | Pole |  |
| Ms4a4a |  | BC055324 |  | AA986860 | YES |
| Pex5l |  | Epb41l4a |  | Cd83 |  |
| Casq2 |  | Il7 |  | Tiparp | YES |
| Ldb3 |  | Rad51ap1 |  | Hspa1a |  |
| Nxpe5 |  | Ccn1 | YES | Cacng7 |  |
| Flrt3 |  | Zfp36 |  | Prelid2 | YES |
| Gdf3 |  | Col6a6 |  | Brca1 |  |
| Gdf15 | YES | Cytip |  | Cidec |  |
| Ms4a14 |  | Tmem100 |  | Tacc3 | YES |
| Trem1 |  | Dlg2 | YES | Rad54b | YES |
| Lmtk3 |  | Lrrc27 |  | Mmp9 | YES |
| Fjx1 |  | Cep55 |  | Ncaph |  |
| Arsi |  | Slamf9 |  | Jdp2 |  |
| Pcdhga10 |  | Gpr132 |  | Clec4a3 |  |
| A530064D06Rik |  | Corin |  | Cd248 |  |
| Fgf7 |  | Zik1 |  | Chrna4 |  |
| Nr4a1 | YES | Shcbp1 | YES | Cnnm4 |  |
| Zbtb8b |  | Foxc2 |  | Gas2 | YES |
| Stc1 |  | Tlr8 |  | Sept6 |  |
| Actc1 |  | Galnt12 |  | Fbxo44 |  |
| Osm |  | Runx2 | YES | Pakap_2 |  |
| 2210407C18Rik |  | Fanci |  | Ect2 | YES |
| Tnfaip6 |  | Maff |  | Adamtsl1 |  |
| Cacna1g |  | Otud1 | YES | Ptpn22 |  |
| Kntc1 |  | Lpar1 |  | Tceal1 |  |
| Tmem139 |  | Gab3 |  | Glt28d2 |  |
| Il1r2 | YES | Sytl2 |  | Fmod |  |
| Ska1 |  | Flnc |  | Klf4 | YES |
| H2-Q1 |  | Prdm1 |  | Gm13889 |  |
| Rasd1 |  | Rem1 |  | Tspan18 |  |
| Kif15 |  | Kif23 |  | St8sia4 |  |
| Gxylt2 | YES | F2rl1 | YES | Pik3cg |  |
| Nat8l |  | Fam222a | YES | Fam102b |  |
| Gadd45b | YES | Cpxm2 |  | Mms22l |  |
| Eno2 |  | Fzd3 |  | H4c9 |  |
| AL935121.1 |  | Twist1 |  | Dpysl3 |  |
| Klc3 |  | Card9 |  | E2f1 | YES |
| Fkbp1b |  | Npnt |  | Oas3 |  |
| Dlgap5 | YES | Camkk1 |  | Smc2 |  |
| Misp3 |  | B9d1 |  | Ift81 |  |
| Retnlg |  | Slc14a1 |  | Il1b |  |
| BC030867 |  | Lat2 | YES | Vars |  |
| Sap25 |  | Mt2 |  | Cttnbp2nl |  |
| Cldn4 | YES | Krt23 | YES | Cbarp |  |
| Mfap2 |  | Gabrb3 |  | Parp8 |  |
| Ncapg |  | Rnf180 |  | Rgs19 |  |
| Adam32 | YES | Large2 |  | Pmepa1 |  |
| Celsr3 |  | Dclk1 |  | Eda |  |
| Ttk |  | Olig1 |  | Hspbap1 | YES |
| Gm5150 |  | Fbn2 |  | Nuak1 | YES |
| Chrm3 |  | Tnfrsf23 |  | Stk17b | YES |
| Themis |  | Pdk3 |  | Plk2 | YES |
| Itgbl1 |  | Arg2 |  | Cyp21a1 |  |
| Abca4 | YES | Ms4a4c |  | Hspa2 |  |
| Tmem28 |  | Tmem178 |  | P3h3 |  |
| Apcdd1 |  | Sorl1 | YES | Arl4a |  |
| Sirpb1b |  | Apba1 |  | Cdon |  |
| Ereg |  | Fam217b |  | Akap12 | YES |
| Cdh15 |  | Lxn |  | Bmper | YES |
| Sgo1 |  | Fignl1 |  | Cd34 |  |
| Car13 |  | Ust | YES | Sccpdh |  |
| Apold1 |  | Kif4 |  | Arl4d | YES |
| Cenph |  | Plin4 | YES | Tagln |  |
| Gm38392 |  | Kif11 | YES | Zfand2a | YES |
| Egr3 | YES | Ptgds |  | Abcb1a | YES |
| Slc44a4 | YES | Nusap1 |  | Clec4a2 |  |
| Ch25h |  | Wfdc2 |  | Ddit3_1 |  |
| Ier2 | YES | Cdca7l |  | Zfp36l1 | YES |
| Dusp1 | YES | Pkia |  | Ifrd1 | YES |
| Ntrk2 | YES | Ccne1 | YES | Ifi211 |  |
| Thsd7a |  | H3f3b |  | Mcm5 |  |
| Abhd1 |  | Gpr65 |  | Dbf4 |  |
| Slc10a6 |  | H2-Q2 |  | Clec1a |  |
| Tox3 | YES | Csrnp1 |  | Chml |  |
| Stil |  | Ptger4 |  | Pdzk1ip1 |  |
| Lca5 |  | Lmcd1 |  | Vcpkmt |  |
| Pkp1 |  | Mid1 |  | Fmnl1 |  |
| H1f10 |  | Pf4 |  | Aqp8 |  |
| Hivep3 | YES | Fam177a |  | Gabra3 |  |
| Ercc6l |  | Ccn2 | YES | Tmem218 |  |
| B4galt2 |  | Ms4a6b |  | Rad9a |  |
| Zbtb9 |  | Igsf3 |  | Ophn1 |  |
| Galnt17 |  | Pask |  | Scd1 |  |
| H2-M2 |  | Gpx8 |  | Dynlt1f |  |
| Sema3b | YES | Ppp1r15a | YES | Mcm3 |  |
| Cd244a |  | Anks6 | YES | Ccdc34 |  |
| Slit3 |  | Orm3 |  | Hes1 | YES |
| Mcub |  | Serpine2 | YES | Nfkbia | YES |
| Exo1 |  | Atp10a |  | Hk1 | YES |
| Polq |  | Epha3 |  | Plk3 | YES |
| Fam83a |  | Spon1 | YES | Abca9 | YES |
| Rad18 | YES | Prkar2b |  | Clstn3 |  |
| Slc25a25 | YES |  |  |  |  |
